# Three-Axis Model for Atg Recruitment in Autophagy against *Salmonella*

**DOI:** 10.1155/2012/389562

**Published:** 2012-02-28

**Authors:** Takeshi Noda, Shun Kageyama, Naonobu Fujita, Tamotsu Yoshimori

**Affiliations:** ^1^Department of Genetics, Graduate School of Medicine, Osaka University, 2-2 Yamadaoka, Suita, Osaka 565-0871, Japan; ^2^Laboratory of Intracellular Membrane Dynamics, Graduate School of Frontier Biosciences, Osaka university, 3-1 Yamadaoka, Suita, Osaka 565-0871, Japan; ^3^Protein Metabolism Project, Tokyo Metropolitan Institute of Medical Science, Tokyo 156-0007, Japan

## Abstract

*Salmonella enterica* serovar Typhimurium enter epithelial cells and take up residence there. Within epithelial cells, a portion of the bacteria are surrounded by an autophagosome-like double-membrane structure, and they are still residing within the *Salmonella*-containing vacuole (SCV). In this paper, we will discuss how the autophagy machinery is recruited in proximity to *Salmonella*. The formation of this double membrane requires Atg9L1 and FIP200; these proteins are important for autophagy-specific recruitment of the PI3-kinase complex. In the absence of Atg9L1, FIP200, and PI3-kinase activity, LC3 is still recruited to the vicinity of *Salmonella*. We propose a novel model in which the mechanism of LC3 recruitment is separate from the generation of the isolation membrane. There exist at least three axes in Atg recruitment: ULK1 complex, Atg9L1, and Atg16L complex.

## 1. Introduction

Autophagy is primarily a process that delivers cytoplasmic component to lysosomes for degradation. In its original definition, autophagy was conceptually paired with phagocytosis; the former is also termed ‘‘autophagocytosis” and the latter ‘‘heterophagocytosis,” although these terms are less frequently used in modern parlance. The two processes can be distinguished according to what the cell ‘‘eats,” namely, itself in the case of autophagy or foreign bodies such as bacteria in the case of phagocytosis. We now know, however, that these processes are not entirely distinct from one another. The molecular apparatus identified during early studies in autophagy turns out also to be involved in the processes associated with the infecting bacteria from the cells of mammals and other organisms such as insects. For example, once *Salmonella enterica* serovar Typhimurium invades nonphagocytic cells such as epithelial cells, a subpopulation of the bacteria becomes decorated with autophagic marker proteins. Many studies have been performed on these phenomena, which some have termed “xenophagy” [1–3]. In this paper, we will discuss the mechanism by which the autophagic machinery is recruited, focusing especially on the case of *Salmonella*.

*Salmonella enterica* serovar Typhimurium, a gram-negative bacterium, infects small intestinal epithelial cells and develops as an intracellular bacterium within this niche, where it causes gastroenteritis [4, 5]. These events allow this bacterium to cause widespread infection. Therefore, in order to better control *Salmonella* infection, it is important to understand the mechanisms by which *Salmonella* develops into an intracellular bacterium in host cells. *Salmonella* possesses a type III secretion system (TTSS), which employs a needle-like structure to inject effector proteins into the host cell's cytosol [4, 6]. By injecting a number of effector molecules into the host cytosol, *Salmonella *can invade epithelial cells via a type of endocytic pathway. Following invasion, the bacteria form a specialized single membrane organelle, the Salmonella-containing vacuole (SCV), by modifying endosomal structures. In the early phase, the SCV temporarily displays early endosome markers, such as Rab5, and EEA1; subsequently, these makers are replaced by late endosomal proteins, such as LAMP1 [7–9]. SCV succsessively develops into a variety of long tubular structure, termed spacious vacuole-associated tubules (SVATs), SNX3 tubules, and the *Salmonella*-induced filaments (Sifs), after a few hours following infection; thereafter, the bacteria can slowly propagate in SCV and the related structures [7–10]. Meanwhile, a subpopulation of *Salmonella* is targeted for xenophagy, beginning with the recruitment of autophagic machinery to the vicinity of the infecting bacteria. In an experimental system using mouse embryonic fibroblasts, 40% of infected *Salmonella *is decorated by autophagic marker proteins one hour after infection [11, 12]. In the absence of autophagic capacity in host cells, the *Salmonella* replicates more extensively [11, 12]. Xenophagy may serve as a backup system to limit the growth of infections in situations in which the SCV is somehow malformed. It was suggested that SCV is damaged by the action of TTSS, resulting in the induction of autophagy toward it [11].

## 2. Role of LC3 in *Salmonella* Xenophagy

The first identified specific marker of the mammalian autophagosome, microtubule-binding protein light chain 3 (LC3) is localized on the autophagosome, or its immediate precursor structure, the isolation membrane. LC3 is also distributed throughout the cytosol. Therefore, cells expressing GFP-tagged LC3 exhibit a punctate fluorescence pattern when autophagy is induced [13]. Although the exact function of LC3 in autophagy remains to be precisely understood, it has been proposed to play a role in selective autophagy. In contrast to nonselective autophagy, which targets general cytosolic materials, the targets of selective autophagy range from organelles such as mitochondria and peroxisomes to large protein complexes. It is generally understood that each target possesses a specific tag, such as Atg32 for yeast mitochondria [14, 15]. Adaptor proteins such as p62/SQSTM, Alfy and Nbr1 recognize the target-specific tags [16–19]. Because it can bind both p62/SQSTM and Nbr1, LC3 has been proposed to be involved in the recruitment of autophagic machinery at the target, [16–20]. However, recent studies of xenophagy against *Salmonella* have led to another interpretation regarding this recruitment mechanism.

LC3 and its paralogues are orthologs of yeast Atg8 [13]; they are ubiquitin-like proteins, but are modified at the carboxyl terminus with phosphatidylethanolamine (PE) instead of proteins [21, 22]. In yeast, Atg7 and Atg3 serve in these process as E1 and E2 enzymes; in their absence, the PE modification does not occur and starvation-induced autophagy is defective [21]. This is also the case in *Salmonella* xenophagy. In Atg7- and Atg3-knockout MEF cells, GFP-LC3 is not recruited to the vicinity of *Salmonella enterica* serovar Typhimurium; as a result, the bacteria replicate overwhelmingly, leading to host cell death [12]. When mouse embryonic fibroblast cells expressing GFP-Atg5 are challenged with *Salmonella*, GFP signal can be observed around some of the intracellular bacteria, in a pattern reminiscent of LC3 [12]. In the Atg3 knockout MEF, the efficiency of GFP-Atg5 recruitment is not significantly different [12]. Therefore, even in the absence of LC3 recruitment, *Salmonella* can be recognized by the autophagic apparatus. A number of adaptor proteins, including p62/SQSTM, NDP52, and optineurin, bind to LC3 and are involved in xenophagy against a variety of bacteria [23–25]. These interactions with LC3 may be more important for functions rather than recruitment of other Atg proteins.

 This notion is further strengthened from other observations. Two reports, including ours, have revealed detailed phenotypes by depleting the function of all LC3 paralogues involved in starvation-induced autophagy. One group knocked out Atg3 genes in mouse embryonic fibroblasts [26]; another exogenously expressed mutant Atg4B [27]. Atg4 is protease involved in the cleavage of the carboxyl termini of nascent Atg8/LC3 family members and their PE conjugates [22]. Overexpression of Atg4B containing a point mutation at its catalytic center aminoacid, cysteine, titrates out LC3 and its homologues by binding them strongly and preventing the PE conjugation reaction [28]. Both approaches yielded essentially the same results: accumulation of incomplete and unsealed autophagosomes in the cytosol [26, 28]. In these experiments, significant proportions of the autophagosome membranes were mostly, but not completely, closed [26, 28]. Yeast Atg8 can catalyze hemifusion of the vesicles with which it associates *in vitro* [29]. Based on these results, we proposed a ‘‘reverse-fusion” model in which LC3 functions in the closing process by directly catalyzing membrane hemifusionmembran- like process [30]. Beyond this role in closing, it remains controversial whether LC3 is involved in the elongation of the autophagosomal membrane [26, 28, 31, 32]. There is also a report that atypical autophagy, which does not require this LC3 system, exists [33]. In mouse embryonic fibroblast cells expressing GFP-LC3, a punctate GFP signal appears in the proximity of infecting *Salmonella* cells and elongates along the surface of the bacterium, just as the isolation membrane elongates to become the autophagosome [12]. Using fluorescence microscopy and electron microscopy correlation, the membrane structure corresponding to GFP-positive *Salmonella* was observed [12]; a double-membrane structure resembling the canonical autophagosome surrounded the *Salmonella* cells. Inside the double-membrane structure, another single membrane thought to be the SCV could also be observed. Therefore, at least in the case of xenophagy in this system, *Salmonella* is surrounded by autophagosome in addition to the SCV [12, 34]. In MEFs lacking Atg7, the E1 enzyme for the LC3 lipidation reaction, a double membrane surrounds the SCV, though it may not be completely closed [12]. Similar images were observed in the MEFs lacking Atg5 [23]. Atg5 is a subunit of the E3 involved in LC3 lipidation, and the mutant is defective in this process [35, 36]. These observations clearly indicated that LC3 function is required neither for membrane elongation nor for recruitment of the autophagic membrane to the target. Therefore, another LC3-independent targeting mechanism must exist.

## 3. Atg9-Independent Recruitment of Atg16L

The next question arising is the identity of the alterative factors that actually do recruit the autophagic machinery. Good candidates for these factors are found among other Atg proteins that function in starvation-induced autophagic process [37]. Atg9 is a six-transmembrane protein, essential for autophagy [38, 39], whose precise role remains to be determined. Mammalian cells have two Atg9 homologues, Atg9L1 and Atg9L2, but the latter is expressed only in placenta and pituitary [40]. In mammalian cells, Atg9 travels around the Golgi and endosome and potentially the autophagosome [39]. Knockout of only Atg9L1 brings about severe defects not only in canonical autophagy but also in *Salmonella* xenophagy, evidenced by observations that *Salmonella* replication dramatically increases within infected Atg9L1- knock out cells, just as in Atg7-knock out cells [12, 41]. Even in Atg9L1-knockout MEF cells, GFP-LC3 is efficiently recruited to *Salmonella enterica* serovar Typhimurium at levels comparable to those observed in wild-type cells [12]; however, in these mutant cells, GFP-LC3-positive *Salmonella* is not surrounded by an autophagosome-like double membrane [12]. Thus, Atg9L1 is required for membrane formation in autophagy, but indispensable for LC3 recruitment. This finding was not anticipated based on results from previous studies. The Atg16L complex consists of two sets of Atg16L1 and Atg12–Atg5 conjugate, bound by a ubiquitination-like reaction [42]. Atg12 binds to Atg3, the E2 enzyme of the LC3 lipidation reaction, and the lipidation reaction occurs where Atg16L is localized [36]. Based on these observations, Atg16L complex serves an E3-like role by linking E2 to the target (PE in membrane) in the LC3 lipidation reaction [36, 43]. The Atg16L complex is exclusively localized on forming autophagosome, the isolation membrane in starvation-induced autophagy [35, 44]. In the case of *Salmonella* xenophagy, however, even in the absence of an autophagosome-like double membrane, Atg16L complex can localize to the vicinity of infecting *Salmonella* [12]. This implies that the Atg16L complex can be recruited to SCV independent of the existence of a double-membrane structure [12]. It remains to be determined whether the same mechanism is also applicable to the wild-type cell, but it is highly likely that some targeting mechanism exists that is independent of both the double membrane and LC3.

## 4. ULK1 Complex Functions in Parallel to Atg9L1

Ulk1 is a mammalian orthologue of yeast Atg1 protein kinase, which is essential for autophagy [45, 46]. Ulk1 forms a protein complex with FIP200, Atg13, and Atg101 [47]. In MEF cells lacking FIP200, *Salmonella* xenophagy is defective, as is starvation-induced autophagy [12]. In the FIP200 knockout, phenotypes pertaining to GFP-LC3 localization and autophagosome-like double-membrane generation were quite similar to those of Atg9L1-knockout cells: GFP-LC3 is efficiently recruited around *Salmonella enterica* serovar Typhimurium, and the double membrane is not observed [12]. One plausible explanation for this result is that one of the proteins is responsible for the recruitment of the other to the vicinity of *Salmonella*, but this is not the case. In FIP200 knock out cells, Atg9 is recruited to *Salmonella*, whereas in Atg9L-knock out cells, Ulk1 is recruited [12]. Thus, localizations of the two proteins are independent of each other. On the contrary, Atg9L1 accumulates to a greater extent near *Salmonella* in FIP200-knock out cells; likewise, Ulk1 accumulates in Atg9L1-knock out cells [12]. Ulk1 complex and Atg9L1 are potentially recycled between the vicinity of *Salmonella* and other cytosolic pools; detachment of either protein from *Salmonella* appears to be dependent on the other. These two players seem to play quite important roles in membrane biogenesis, and it is likely that their functions are tightly coupled with their recycling. There are both similarities and differences between these models and what has been observed in yeast autophagy. In yeast, Atg1 (Ulk1 homologue) is also required for recycling of Atg9 from the PAS, the site of autophagosome formation, to other pools [48]. However, targeting of Atg9 to the PAS is dependent on Atg17, a potential counterpart of FIP200, through direct binding [49]. This FIP200-independent Atg9l1 localization may be explained by the fact that a part of Atg9L is transiting early endosome, which is closely associated with SCV at steady state, even in the absence *Salmonella* infection [12].

## 5. PI3P Involvements in *Salmonella* Xenophagy

**Figure 1:**
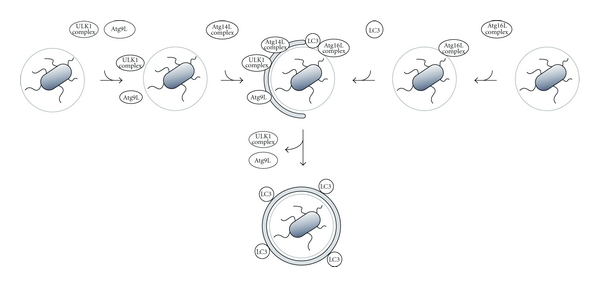
Schematic representation of the three-axis model for Atg recruitment in *Salmonella* xenophagy *Salmonella enterica* serovar Typhimurium is inside the SCV, but some bacterial cells are captured by the autophagic machinery. LC3 is recruited by Atg16L complex, but this recruitment is not dependent on the other factors depicted here. Even in the absence of these factors, an autophagosome-related membrane is observed. Ulk1 complex (including FIP200) and Atg9L1 recycle between the vicinity of *Salmonella* and the other cellular pools. Both are recruited to *Salmonella* independent of one another, but their detachment from *Salmonella* proximity is interdependent. Atg14L-containing PI3-kinase complex recruitment is dependent on both Ulk1 complex and Atg9L1.

PI3P plays critical roles in canonical starvation-induced autophagy [50].When cells are treated with wortmannin, a potent inhibitor of PI3-kinase, LC3 localization to autophagosome is completely defective [13]. In the case of *Salmonella* xenophagy, however, wortmannin treatment does not affect LC3 targeting to the vicinity of *Salmonella enterica* serovar Typhimurium [12], although another study showed some reduction in the efficiency [51]. This does not necessarily mean, however, that PI3P is indispensable for *Salmonella* xenophagy. For starvation-induced autophagy, there exists a specific PI3-kinase protein complex, consisting of Vps34, Vps15, Beclin-1, and Atg14L [52–54]. The knockdown of Atg14L, the sole complex-specific subunit, leads to *Salmonella* overgrowth in infected cells [53]. The localization of Atg14L is also observed in proximity to infected *Salmonella* [12]. WIPI-1, a PI3P-binding protein involved in autophagy, is also observed there, and this localization is sensitive to wortmannin treatment [12]. Thus, similar to the case of Atg9L1 and Ulk1 complexes, autophagy-specific PI3-kinase activity is involved in *Salmonella* xenophagy, but is dispensable for LC3 targeting [12]. This is easily understandable in light of the fact that localization of Atg14L to *Salmonella* becomes defective in cells lacking either Atg9L1 or FIP200 [12]. This implies that both Atg9L1 and Ulk1 complexes are upstream determinants of autophagy-specific PI3-kinase localization. In the case of starvation-induced autophagy, autophagy-specific PI3-kinase is targeted to the endoplasmic reticulum, where it forms foci (the “omegasome”) in order to form the autophagosome [54]. This omegasome is marked by DFCP-1 through its PI3p-binding capacity, whose function in autophagy is still unclear [55]. DFCP-1 is closely associated with *Salmonella* xenophagy, so this may take place in close proximity to the ER [51] (see Figure 1).

## 6. Conclusion

It is now clear that there exist at least three independent axes for the recruitment of autophagic machinery to the vicinity of *Salmonella enterica* serovar Typhimuriuma*:* Atg16L complex, Atg9L1, and Ulk1 complex. In the case of yeast autophagy, Atg17, a subunit of Atg1 complex, is proposed to be a fundamental determinant of the recruitment of other Atg proteins to the PAS [56]. In the case of mammalian starvation-induced autophagy, a similar role has been proposed for FIP200 [57]. However, both cases of starvation inducue autophagy lack the existence corresponding to SCV, which can become an alternative membrane target of Atg16L complex (i.e., instead of the autophagosome). Therefore, the possibility that an Ulk1 complex-independent Atg16L recruitment mechanism is also involved in starvation-induced autophagy cannot be eliminated.

In that case, what factors exist upstream of Atg16L and Ulk1 complexes? Involvement of adaptor proteins is highly likely, although their direct binding to LC3 is indispensable. Ubiquitin and several adaptor proteins are recruited to the vicinity of *Salmonella*, so they must play critical roles [23–25]. It is possible that these adaptor proteins also bind other Atg proteins, such as Ulk1 and Atg16L complexes. In this regard, it is noteworthy that Tecpr1, a novel adaptor protein involved in xenophagy, binds to Atg5 [58]. Combining with other important players such as diacylglycerol [59], understanding the direct trigger for *Salmonella* xenophagy represents the next important step for this field.

## Abbreviations

MEF: Mouse embryonic fibroblast
S. Typhimurium: Salmonella enterica serovar Typhimurium
PI3K: Phosphatidylinositol 3-kinase
ULK: Uncoordinated 51-like kinase
FIP200: FAK family: interacting protein of 200 kD
WIPI: WD-repeat protein interacting with phosphoinositides
PAS: Preautophagosomal structure
SCV: Salmonella-containing vacuole.
